# Sustainable integrative cell biology: CENP-C is guilty by association

**DOI:** 10.1007/s10577-025-09784-0

**Published:** 2025-11-25

**Authors:** Natalia Y. Kochanova, Itaru Samejima, William C. Earnshaw

**Affiliations:** Institute of Cell Biology, The University of Edinburgh, Edinburgh, Scotland EH9 3BF UK

**Keywords:** Cell biology, Centromeres, Discovery of CENPs, Correlation, Guilt by association

## Abstract

In the 40 years since the discovery of the CENP proteins, many studies have examined the role of these proteins and their interactions with other chromosomal proteins of the centromere and beyond. Together, these studies have yielded vast amounts of sequencing and proteomics data. Typically, each study has focused on a single question and the majority of each dataset remains largely unexplored. Often the interesting details of publicly deposited data are left behind, buried in archives online, while more and more new data are generated. Reanalysing these databases can represent a new paradigm for investigating diverse biological pathways in unprecedented detail. Here, we explore two publicly available pan-cancer proteomic datasets to compare proteins whose abundance correlates with CENP proteins, with a particular focus on CENP-C. Our analysis confirms an expected link between CENP-C and cohesin levels but reveals a surprising and unexpected correlation between CENP-C and proteins of the inner nuclear membrane and the NuMA protein. This guilt-by-association analysis has the potential to identify proteins that act in common pathways but never associate or colocalize and may not even be expressed at the same time in cells. As an example, we show here that it can reveal unexpected links that expand our conception of centromeric chromatin beyond chromosome segregation.

**Supplementary Information:**

The online version contains supplementary material available at 10.1007/s10577-025-09784-0.

## Introduction

### Brief history of findings arising from ACA

In 1984 we collaborated with Dr. Naomi Rothfield to identify the human autoantigens recognised by anti-centromere antibodies (ACA), which had been first described by Moroi, Tan and co-workers (Moroi et al. 1980). Taking advantage of her bank of some 80,000 patient sera stored at −80 °C, we identified several patterns of antigens recognised when isolated human mitotic chromosomes were used as substrate in immunoblots. The most commonly recognised antigens were given the name CENPs (CENtromere Proteins), A, B and C (Earnshaw and Rothfield 1985). Every patient diagnosed with ACA had antibodies to CENP-B, sometimes at an astoundingly high titer (Earnshaw et al. 1987a). Antibodies to CENP-C were also common, but not ubiquitous in these sera. We did not determine the prevalence of anti-CENP-A because the blot detection system had nonspecific background staining of the core histones and this overlapped with the specific recognition of CENP-A. We referred to these antigens by the family name of CENP because antibodies affinity purified from CENP-B also cross-reacted with CENPs A and C. Antibodies affinity purified from CENP-C cross-reacted only with CENP-B (Earnshaw and Rothfield 1985). CENPs A, B and C do not share common amino acid sequence motifs and the explanation for this cross-reaction remains unknown.

Also present in a subset of these sera were antibodies to a group of proteins that migrated in SDS-PAGE with apparent molecular weights of 20–25 kDa. cDNA cloning revealed that at least one of these proteins was related to Drosophila heterochromatin protein HP1 (Saunders et al. 1993). We therefore proposed naming the proteins HP1α, HP1β, and HP1γ. Research on HP1α since then has emerged into a separate large field, investigating the interplay of factors in H3K9me2/3-containing heterochromatin (Bell et al. 2023), but as far as we are aware, little has been subsequently done with these autoantibodies to HP1s.

Following the identification of the three CENP antigens, the obvious next step was to try to figure out what other proteins they interacted with. Unfortunately, this is where our initial investigations hit a brick wall. In order to do a pull-down or immunoprecipitation of a target protein, the first requirement is that the protein must be soluble. However, every method that we initially used to try and solubilise the proteins failed. Indeed, it appeared that the CENP antigens were all part of the chromosome scaffold fraction—the most insoluble proteins of mitotic chromosomes, which Uli Laemmli had proposed to form a network responsible for giving chromosomes their characteristic shape (Adolph et al. 1977; Paulson and Laemmli 1977; Laemmli et al. 1978; Earnshaw et al. 1984).

A subsequent study from our group tried a different approach to find proteins capable of binding to CENP-C. This affinity chromatography study used CENP-C expressed in E. coli that was bound to a column and asked whether proteins from HeLa cell extracts could bind to it. This study yielded two binding partners: UBF—an essential transcription factor that binds to ribosomal gene promoters (Pluta and Earnshaw 1996), and DAXX—a histone chaperone (Pluta et al. 1998). Accordingly, ectopic mislocalization of CENP-A was shown to be dependent on DAXX (Lacoste et al. 2014), and DAXX promotes proper CENP-A levels upon replication (Lee et al. 2025) and DNA damage (Trier et al. 2023) and associates with CENP-B (Morozov et al. 2017).

The comprehensive identification of proteins located around the CENP antigens came only a number of years later, when experiments from the Yoda and Cleveland labs used nucleases to digest chromosomes to oligonucleosomes and then then do pulldowns with antibodies to CENP-A. The study from the Yoda group yielded a set of proteins that they termed the ICEN (Interphase Centromere Network (Izuta et al. 2006)). The Cleveland group study yielded the seven-protein CENP-A nucleosome-associated complex (CENP-A NAC) plus the 7 additional proteins of the CENP-A-nucleosome distal complex (CAD) (Foltz et al. 2006). Independently, Cheeseman and Desai identified a network of 10 interacting proteins in C. elegans (Cheeseman et al. 2004).

At the end of these studies an association of 16 proteins had been identified that was subsequently named the CCAN (Constitutive Centromere Associated Network – (Cheeseman and Desai 2008)). This group of proteins comprises several closely associated complexes that, together with the CENP-A nucleosome, appears to comprise the heart of the kinetochore. The functions of these proteins and interactions between them have been the subject of many elegant papers over the years from the Fukagawa lab, largely exploiting the power of gene targeting in chicken DT-40 cells (Okada et al. 2006; Hori and Fukagawa 2012; Fukagawa and Earnshaw 2014; Nagpal and Fukagawa 2016; Ariyoshi and Fukagawa 2023). Building upon initial studies from the Brinkley lab (Brenner et al. 1981; Brinkley et al. 1986; He and Brinkley 1996), their distribution has been examined by immunoelectron microscopy (Cooke et al. 1990, 1997; Saitoh et al. 1992; Suzuki et al. 2011; Chen et al. 2025) and super-resolution microscopy (Joglekar et al. 2006, 2009; Wan et al. 2009; Sacristan et al. 2024). The structure of the interphase form of the CCAN complex has been solved at near-atomic resolution (Yatskevich et al. 2022; Pesenti et al. 2022; Dendooven et al. 2023).

We now know that at the base of the kinetochore is a nucleosome containing CENP-A, a variant form of histone H3 (Palmer et al. 1991; Sullivan et al. 1994). Associated with this nucleosome are several complexes, including one containing the proteins CENPs-T, -W, -S—X that also makes a histone-like fold and coordinates one turn of the centromeric DNA (Nishino et al. 2012). Holding the entire network of 16 proteins together is an extended molecule of CENP-C, which weaves through the complex and also makes important contacts tethering the outer microtubule-binding components of the kinetochore to it (Przewloka et al. 2011; Screpanti et al. 2011; Klare et al. 2015).

Associated with this complex is one of two outliers—CENP-B (more on the other outlier—CENP-E—later). CENP-B is not an integral part of the inner kinetochore. Instead, it is the only centromere protein in humans that binds to a specific DNA sequence, as initially predicted based on monoclonal antibody reactivity (Earnshaw et al. 1987b) and later elegantly demonstrated by Masumoto et al. (Masumoto et al. 1989; Muro et al. 1992) to bind to the 17 bp CENP-B box sequence in its unmethylated state. The role of CENP-B remains enigmatic. The protein is encoded by an unusual intron-less gene and has two extraordinary acidic regions (Earnshaw 1987). CENP-B appears to be derived from a transposase of the Marriner family (Earnshaw et al. 1987b; Tudor et al. 1992), but it lacks any detectable enzymatic activity. CENP-B can promote the formation of both heterochromatin and euchromatin (Otake et al. 2020), and it appears to be essential for the formation of human artificial chromosomes in human cells (Ohzeki et al. 2002, 2020; Okada et al. 2007). The molecular function of CENP-B is not known, but it does appear to promote CENP-C association with chromatin and thereby promote kinetochore assembly (Fachinetti et al. 2013, 2015; Hoffmann et al. 2016).

The presence of CENP-B does not necessarily correlate with centromere activity, as the protein is readily detected at the inactive centromere of dicentric autosomes (Earnshaw et al. 1989), though not at the inactive centromere of a dicentric (and therefore inactive) human X chromosome (Earnshaw and Migeon 1985). This latter observation led to the first suggestion that kinetochore assembly might be regulated by an epigenetic mechanism (though the term used was “chromatin conformation” as the use of “epigenetic” to describe histone modifications was not yet in use) (Earnshaw and Migeon 1985). This explanation of the CENP-B binding was later confirmed when Masumoto and co-workers showed that methylation blocks CENP-B binding to the CENP-B box (Tanaka et al. 2005; Okada et al. 2007). The role of CENP-B binding to CENP-B boxes remains enigmatic (Dubocanin et al. 2025).

Interestingly CENP-B is not found at all centromeres. In humans, the Y chromosome lacks CENP-B boxes in its α-satellite DNA array and CENP-B protein does not bind to it (Earnshaw et al. 1991; Fachinetti et al. 2015). Bizarrely, African green monkey (the species in which α-satellite was first identified (Musich et al. 1980)) has CENP-B protein, but no CENP-B boxes in its α-satellite, so CENP-B is not located at centromeres (Goldberg et al. 1996; Kasinathan and Henikoff 2018).

### Probing functional interactions for centromere proteins

In 2013, we proposed “forensic integrative cell biology” as an approach to deduce novel protein function in the post-genomic era, through the detailed analysis of previously published multi-omics data (Earnshaw 2013). Here, using this approach we generate new hypotheses linked to centromere biology based on high-throughput proteomic studies. In view of the fact that our approach builds on the re-use of previously published data-sets, we propose to alter the term to relate more to current usage and will refer to the approach as “sustainable integrative cell biology”.

Now that the components of the core kinetochore are largely identified, our interest has turned to characterising other components that associate with centromeres and contribute to their special properties in directing mitotic chromosome segregation. Over the years, many proteins have been shown to be associated with centromeres. For example, a biotinID study from our group used CENP-A as a bait and found a number of known centromere-associated proteins in proximity to it (Remnant et al. 2019).

Here, we describe an orthogonal approach that is not based on direct physical association or proximity in space. Instead, this approach—a form of guilt-by-association (GBA)—looks at many thousands of proteins by mining two published datasets. One, a comprehensive proteomics characterisation of the proteome of 949 cancer cell lines, includes some 8,498 proteins (Goncalves et al. 2022). The second consists of a set of 15,312 protein groups across 1172 samples derived from 999 patient samples from 22 cancer types plus quality controls (Knol et al. 2025). Our method selects a protein of interest and then looks across these datasets for the other proteins whose abundance most closely correlates with it.

Our study was inspired by a classic study from Walker in 2001 (Walker 2001). He gained access to 1,176 cDNA libraries in the LifeSeq database, sequenced roughly 5,000 cDNAs from each library, and aligned the resulting > 37,000 sequence fragments against known genes. His analysis assumed that the abundance of individual cDNAs correlates with the abundance of the corresponding mRNAs in the target cells. He decided to use this approach to look for novel (e.g. unknown) proteins involved in the cell cycle. He therefore interrogated his database looking for all cDNAs that correlated with a group of target proteins that he designated as markers of cell division. This analysis yielded a group of 8 cDNAs which he called CDCA1-8 (Cell Division Cycle). The "hit rate" of this analysis was remarkable. When he did this, he had no idea what was encoded by those cDNAs. Today, we know that CDCA1 is Nuf2, a key outer kinetochore component of the NDC80 complex. CDCA2 is RepoMan, a mitotic regulator of protein phosphatase 1. CDCA3 is an Fbox protein that is required for mitotic entry. CDCA4 is reported to function in the E2F/Rb pathway (Hayashi et al. 2006) and to localise similar to NuMa (Wang et al. 2008). CDCA5 is sororin, an important regulator of the cohesive cohesin complex. CDCA6 is CBX2, which recruits the PRC1 complex to mitotic chromosomes. CDCA7 is an activator of the nucleosome remodeler HELLS that senses hemimethylated CpG and promotes DNA methylation in heterochromatin (Jenness et al. 2018; Funabiki et al. 2023; Wassing et al. 2024). CDCA8 is borealin/Dasra, an essential component of the chromosome passenger complex (Gassmann et al. 2004; Sampath et al. 2004). This is clearly an astounding "hit rate" for proteins that turned out to have important roles in cell division considering the unbiased nature of the initial screen.

As shown by the results of Walker's analysis, GBA appears to be a method for identifying cellular processes and not specific protein complexes, neighbourhoods or specific pathways. Thus, GBA provides a computational way to analyse relationships that effectively complements GO analysis.

We decided to interrogate our source datasets in two ways. First, we selected a target protein of interest—in this case, our pet protein was CENP-C—and asked of the many thousands of proteins in the datasets, what were the top 20 or 25 proteins that correlated with it. Note—we did not set a significance cutoff for these correlations. Instead, we ranked all proteins and looked for the top 20/25 of the thousands of proteins in the dataset. Our hypothesis was that if we analysed huge datasets, even low-level correlations, might reveal proteins that work together in common pathways. We note the importance of ranking the correlations for a particular protein in our method. The initial study of (Goncalves et al. 2022) published the Pearson correlations for many proteins, however all these correlations were merged into one large resource without assigning importance to particular pathways.

In a second analysis, we took all proteins implicated in the CCAN/kinetochore and analysed their correlations within the datasets.

Our analysis yields provocative results that open the door for further biochemical and cell biology investigations. Firstly, we find reproducible association of CENP-C protein levels with the cohesin complex, lamin B1, LAP2α and NuMA. An association of cohesin with centromeres is not surprising (Blat and Kleckner 1999; Megee et al. 1999; Tanaka et al. 1999; Waizenegger et al. 2000; Yeh et al. 2008; Paldi et al. 2020; Haase et al. 2025). Indeed, a recent study reported that cohesin depletion in *Xenopus* egg extracts impairs CENP-C binding to mitotic centromeres (Haase et al. 2025). However, the association with the nuclear lamina and NuMA is unexpected. These results suggest that probing these datasets with proteins of interest offers a powerful novel approach to discovering functional pathways and cellular states. We emphasise that our approach of sustainable integrative cell biology is not intended to yield mechanistic insight or map protein–protein interactions. Instead it can generate hypotheses for further mechanistic analysis by suggesting previously unexpected functional correlations between proteins of interest.

## Materials and methods

### Correlation analysis

The greater part of this analysis was performed using R (R Core Team 2022). In the tables with protein abundances from (Goncalves et al. 2022) and (Knol et al. 2025) we imputed zeros in place of NAs to allow Spearman correlations between all vectors each representing protein abundances across cell lines or patient samples to be calculated. Zeros were imputed instead of NAs in the correlation matrix as well.

The IDs from both pan-cancer datasets were mapped to GO-terms through Uniprot and the median of all correlations between proteins related to each GO term was calculated (excluding correlations equal to 1). The distribution of medians was plotted with the “ggridges” package (Wilke 2024). Synthetic lethal interactions were filtered similar to (Liskovykh et al., submitted): as those with the highest reliability identified by low throughput or CRISPR studies (SynLethDB 2.0 database) (Wang et al. 2022). Correlations between such synthetic lethal interactions were plotted against all correlations in the datasets with “ggridges”.

The correlation matrix was subsetted and plotted for CENPs present in the datasets (except CENP-F and CENP-J) and the NDC80 complex. Correlations and protein abundance ranges were plotted for these proteins as well. The correlations were ordered in a descending order for CENP-C for both datasets. The plots were assembled with ggplot2 (Wickham 2016). The code is available at Github via https://github.com/NatashaKochanova/Correlation-analysis-centromere

## Results and discussion

A major goal of innumerable cell biological studies over the last several decades has been mapping structural and functional relationships between proteins. This involves a huge range of approaches. Modern cell biology began with co-fractionation on sucrose gradients (Deter and de Duve 1967) but has since expanded to occupy a vast experimental range. A non-comprehensive list of approaches includes co-fractionation on various types of columns, co-localization by fluorescence microscopy, proximity analysis by other microscopy methods, including FLIM-FRET and proximity ligation (PLA), yeast two-hybrid screening, pulldowns using specific antibodies or proteins either singly or multiply tagged (analysed either in cell extracts or in vivo), or use of AlphaFold Pulldown. A conceptual breakthrough came with the development of biotin-ID (Roux et al. 2012), which can identify proteins located in the vicinity of a target protein, even if those proteins do not directly interact.

Another type of approach is even broader yet – looking for proteins that function in common pathways. These proteins need not interact, be physically close to one another or even function at the same time. They might simply carry out sequential roles in a common pathway. The classic approach to this is genetic analysis, e.g. using either complementation or synthetic lethality screens (Novick et al. 1980; Bender and Pringle 1991). A computational analogue of this is GO analysis, which exploits curated information about all observations concerning a protein. But a more focused bioinformatic approach is guilt-by-association (Walker et al. 1999; Jin et al. 2008). With the advent and wealth of RNA-seq data, correlation analysis, a particular way of performing guilt-by-association analysis, has been applied to many gene networks (Hong et al. 2013; Sanchis et al. 2021). Indeed, genes, similarly expressed with the target genes, are frequently ranked in databases referring to RNA-seq and CRISPR datasets, as, for example, in the Human Protein Atlas and DepMap (Uhlen et al. 2010; Dempster et al. 2019; Karlsson et al. 2021; Pacini et al. 2021; Arafeh et al. 2024). However, it is important to note, that the reported correlation between mRNA and protein levels in cells is only approximately 0.4 (Schwanhausser et al. 2011). This suggests that guilt-by-association analysis of available pan-cancer proteomic datasets could be more representative of the levels of proteins within cells and might yield different patterns when comparing protein correlations with transcript analysis.

In this MS we use data-mining to demonstrate the ability of guilt-by-association to generate novel hypotheses relating to protein structure and function. Our interest in this began with the classic study by Walker (Walker 2001) in which he used DNA sequencing to analyse the abundance of cDNAs in over a thousand cDNA libraries. Here, we performed rank-based correlation analysis. We present two simple uses of this analysis. We emphasize that this analysis can be conducted for any protein that appears in any large dataset.

### Related proteins correlate more strongly than bulk proteins in proteomics datasets

We performed a guilt-by-association (GBA) correlation analysis (Walker 2001) by mining published pan-cancer proteomic data. We analysed two large cancer datasets. One of them contained measured levels of 8,498 proteins across 949 cell lines (Goncalves et al. 2022). The other one contained abundances of 15,312 protein groups across 1172 samples, (Knol et al. 2025). We calculated the Spearman correlation of protein abundances for every protein versus every other protein in these datasets.

**Fig. 1:**
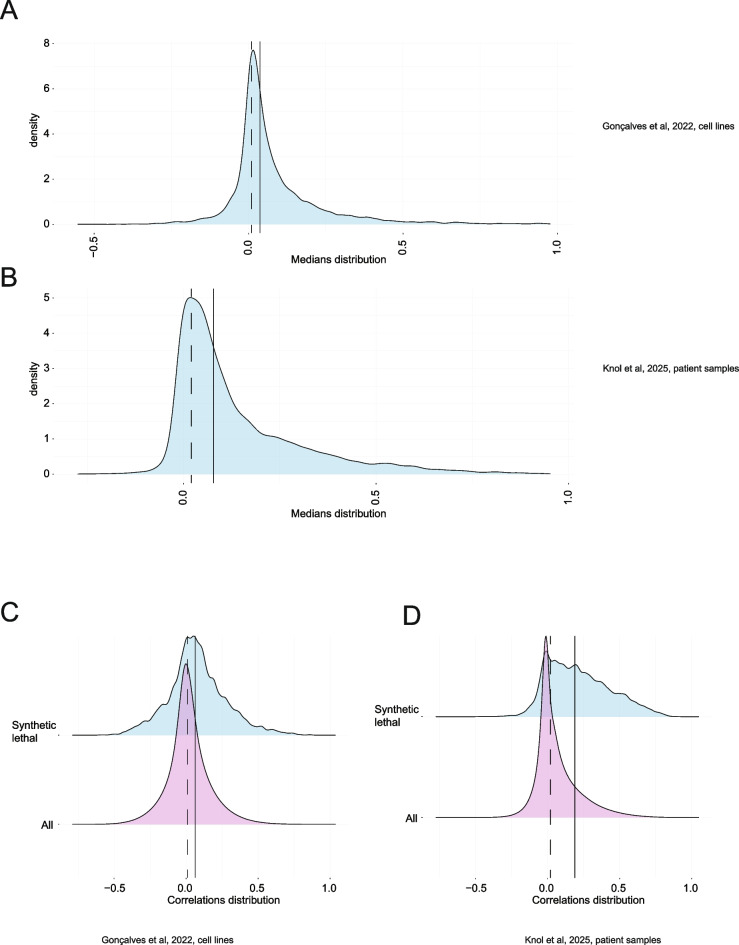
**A**, **B**. Distribution of medians for all correlations between proteins related to each GO term. The median of these medians is plotted as a solid line, while the median of all correlations in a particular dataset is plotted as a dashed line. **C**, **D**. Distribution of correlations between proteins, which are synthetic lethal (blue) vs the distributions of all correlations in the dataset (pink). The median for correlations between synthetic lethal proteins is plotted as a solid line, while the median of all correlations in the dataset is plotted as a dashed line

To validate this guilt-by-association approach, we enquired whether proteins involved in the same pathway tend to correlate more strongly than bulk proteins in the same datasets. We calculated a median for each set of correlations between proteins related to a particular GO term, and plotted the distribution of those medians (Fig. 1A, B). The median of these medians was shifted towards the positive direction in the analysis of both datasets, compared with the median of all correlations in the particular dataset. Thus, proteins within a GO set tend to be more positively correlated to one another than bulk proteins from the over-all dataset.

As an orthogonal approach, we filtered highly confident synthetic lethal interactions between genes (Wang et al. 2022), and plotted correlations between respective proteins as a distribution, compared to all correlations in the dataset (Fig. 1C, D). The median of correlations between proteins that are synthetic lethal was shifted towards the positive direction in the analysis of both datasets. This was especially evident in the analysis of the (Knol et al. 2025) pan-cancer proteome.

This computational analysis points to a trend of correlations being more positive between proteins that are functionally related in cells.

### Ranking of correlations suggests a functional interaction between cohesin and CENP-C

For CENP-A, -B and -C we ranked the correlations in descending order (from positive to negative).

We began by using CENP-C as a query. Our correlation analysis for the cancer cell line dataset revealed that among the top 15 of 8,498 proteins correlating with CENP-C were the three cohesin subunits RAD21, SMC1 and SMC3 (Jachymczyk et al. 1977; Strunnikov et al. 1993; Michaelis et al. 1997; Prevo and Earnshaw 2024; Dekker and Mirny 2024) and boundary factor CTCF, which positions loop-extruding cohesin to form TADs (Topologically Associated Domains) (Lobanenkov et al. 1990; Dekker and Mirny 2024) (Table 1, highlighted in yellow). Our analysis of the second completely independent patient sample-derived dataset found cohesin subunits RAD21, SMC1 and SMC3 (Table 2, highlighted in yellow) and cohesin loader NIPBL (Alonso-Gil and Losada 2023; Prevo and Earnshaw 2024) in the top 25 correlations among the 15,312 protein groups. In this second analysis CTCF ranked at position 729/15,312. These positive correlations suggest that CENP-C and cohesin might function in the same pathway.

**Table 1:**
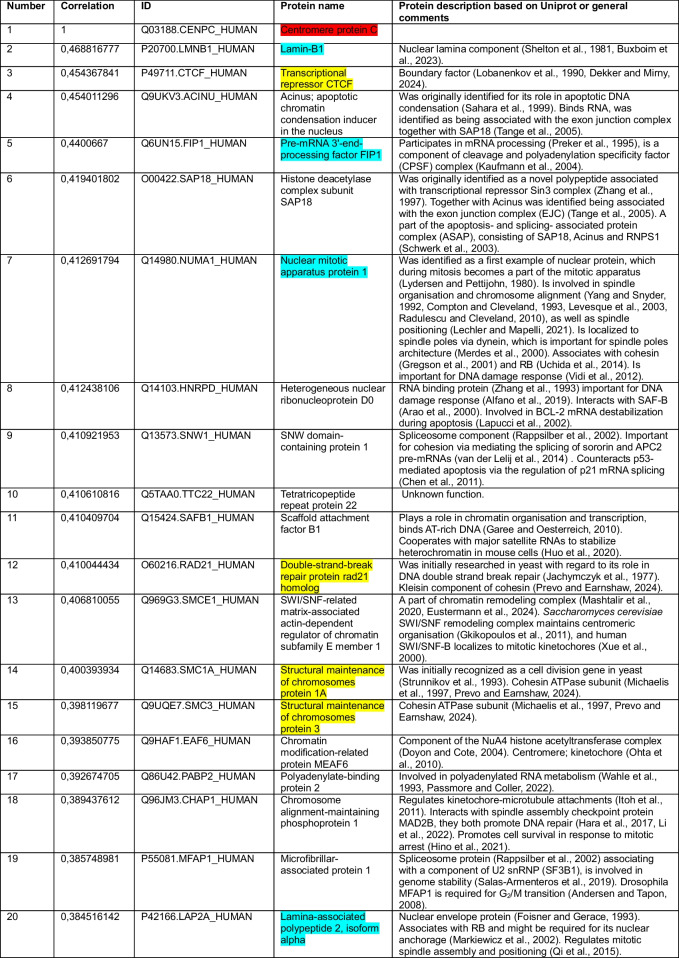
Top-20 CENP-C correlations from a pool of 8,498 proteins measured in (Goncalves et al. 2022)

Color key: CENP-C (red), cohesin and CTCF (yellow), common proteins between two datasets in top-20 or top-25 (blue)

**Table 2:**
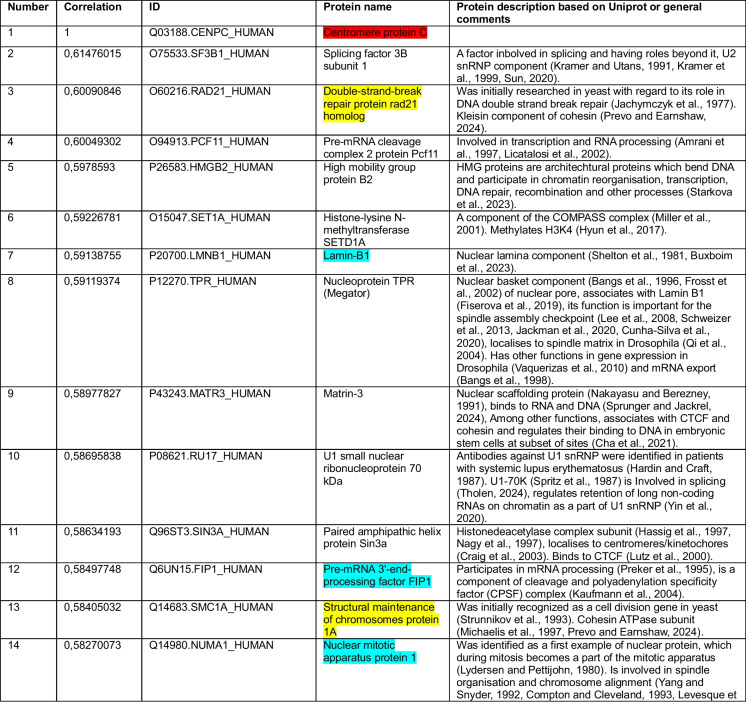

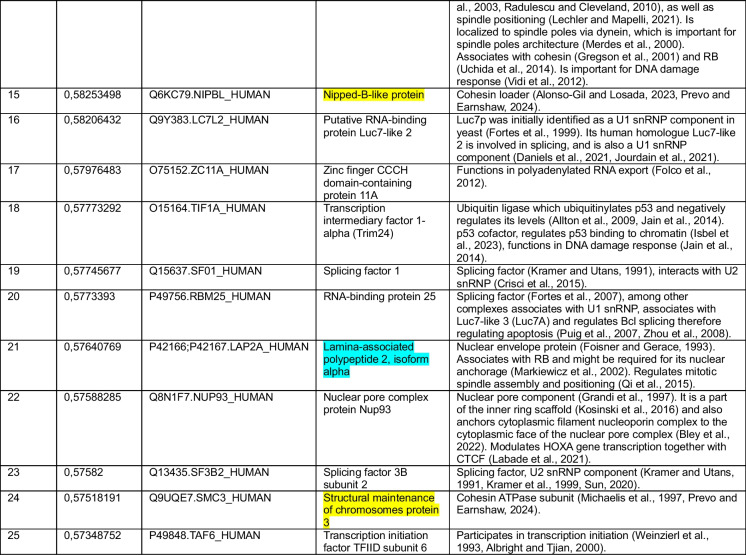
Top-25 CENP-C correlations from a pool of 15,312 protein groups measured in (Knol et al. 2025)

Color key: CENP-C (red), cohesin and NIPBL (yellow), common proteins between two datasets in top-20 or top-25 (blue)

**Fig. 2:**
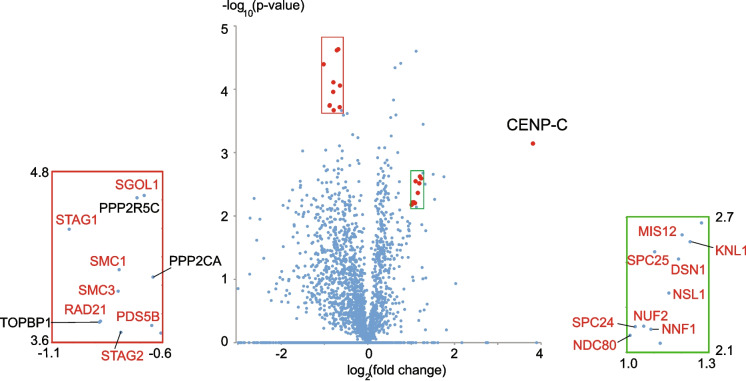
A volcano plot of data from four replicates of the proteome of chromosomes isolated from CENPC^off^ and wild type cells as described in (Samejima et al. 2015). Kinetochore components whose levels decreased upon CENP-C loss are shown in the box at the right. Cohesin components and related proteins whose levels on chromosomes rose following CENP-C depletion are shown in the box at the left. Proteins whose names are indicated in the blow-ups are indicated by red dots in the volcano plot

The link between CENP-C and cohesin is supported by re-analysis of data from a previous proteomics study in which we examined the effect of deletion of various kinetochore proteins on levels of proteins associated with isolated mitotic chromosomes (Samejima et al. 2015). Previously, we reported that CENP-C depletion causes a decrease in levels of members of the KMN network on the chromosomes. We therefore speculated that CENP-C and CENP-T function in parallel in kinetochore assembly. A recent re-analysis revealed that CENP-C depletion correlated with a statistically significant 1.74-fold increase in the levels of cohesin subunits associated with chromosomes (Fig. 2). This observation suggested that CENP-C somehow regulates cohesin function and accumulation at mitotic centromeres – possibly by locally facilitating the action of the prophase pathway of cohesin removal (Sumara et al. 2000).

Other studies – particularly in the budding yeast have described the association of cohesin with centromeres (Yeh et al. 2008; Hinshaw et al. 2017; Paldi et al. 2020). Cohesive cohesin links sister chromatids at centromeres, as well as along chromosome arms (Tanaka et al. 1999), and is important for 3D organization of DNA folding in mitotic chromosomes (Samejima et al. 2025). In budding yeast, loading onto centromeres is achieved via a dedicated receptor, Ctf19 (Hinshaw et al. 2017). Yeast cohesin has been shown to organize a loop architecture that is responsible for mechanical properties of the centromere (Yeh et al. 2008; Paldi et al. 2020). Less was known about the detailed roles of cohesin at metazoan centromeres and as a working hypothesis we speculated that the correlation might reveal a link between CENP-C function (e.g. kinetochore assembly) and cohesin loading. However, a very recent paper now finds that cohesin is responsible for stabilizing the CCAN through CENP-C (Haase et al. 2025). Thus, in this case our correlation analysis extends and confirms experimental data.

Among investigated centromeric proteins, CENP-C was not the only protein clearly exhibiting a positive correlation with cohesin (Supplementary Table S1). In the (Goncalves et al. 2022) dataset, CENP-H, CENP-K, CENP-P, CENO-Q, CENP-U, CENP-X and CENP-V, exhibited top-60 or more highly ranked correlations with selected cohesin subunits, the strongest correlator being CENP-U (SMC1A, SMC3 and RAD21 in its top-10 correlating proteins). In the (Knol et al. 2025) dataset analysis, CENP-B correlated with cohesin, SMC1A being ranked 32, and SPC24 correlated with cohesin as well, SMC1A being ranked 60. Thus, our results are consistent with a potentially broader role for cohesin at centromeres than simply a relationship with CENP-C (Haase et al. 2025).

Other top-ranked proteins correlating with CENP-C included several involved in various aspects of chromatin and cell division (Tables 1 and 2). In addition to cohesin, these include proteins reported to reside at pericentromeric DNA/centromeres/kinetochores (SAFB1 (Huo et al. 2020), SIN3A (Craig et al. 2003), EAF6 (Ohta et al. 2010)) and proteins more generally associated with mitosis (CHAP1 (Itoh et al. 2011), NuMA1 (Lydersen and Pettijohn 1980; Compton and Cleveland 1994; Kiyomitsu and Boerner 2021), TPR (Megator) (Bangs et al. 1996; Qi et al. 2004; Lee et al. 2008; Schweizer et al. 2013; Jackman et al. 2020; Cunha-Silva et al. 2020)). Other proteins correlating with CENP-C included several important for chromatin architecture: cohesin, HMGB2 (Starkova et al. 2023), MATR3 (Nakayasu and Berezney 1991; Sprunger and Jackrel 2024), SAFB1 (Garee and Oesterreich 2010)), several RNA splicing components and histone deacetylase SAP18 (number 6, Table 1) which was shown to be involved in a complex with apoptotic condensation inducer Acinus (number 4, Table 1) (Zhang et al. 1997; Sahara et al. 1999; Tange et al. 2005; Singh et al. 2010). These could be explored in the future to test their functional associations with CENP-C.

### Surprising correlation of CENP-C with components of the nuclear lamina and mitotic spindle

In addition to cohesin, both independent CENP-C correlation lists contained 4 other proteins among the top 20/25 (Tables 1 and 2, highlighted in light blue): Lamin B1 (Shelton et al. 1981; Buxboim et al. 2023), Lamina Associated Protein α (LAP2α) (Foisner and Gerace 1993), Nuclear Mitotic Apparatus-Associated protein (NuMA), and Factor Interacting with PAPOLA and CPSF1 (FIP1) (Preker et al. 1995). The reproducibility of these top correlations between the two independent datasets analyzed strengthens the potential links of these factors with CENP-C.

We know of no existing hypothesis to explain an association between the kinetochore component CENP-C and the nuclear envelope. Previous studies had shown that other kinetochore-associated components – the spindle assembly checkpoint (SAC) proteins MAD1 and 2 – are associated with the nuclear envelope during interphase, and nuclear pore component TPR, also identified in our correlation analysis, is necessary for proper kinetochore levels of Mad1 in prometaphase (Lee et al. 2008; Schweizer et al. 2013; Jackman et al. 2020; Cunha-Silva et al. 2020). During prophase, chromosomes typically condense on the inner surface of the nuclear envelope, so these associations could conceivably be involved at that stage, but this remains a question for further study.

The other two proteins exhibiting high correlation with CENP-C in both lists are NuMA and FIP1. NuMA is involved in spindle organization and chromosome alignment as well as organizing the spindle poles (Yang and Snyder 1992; Compton and Cleveland 1993; Levesque et al. 2003; Radulescu and Cleveland 2010; Kiyomitsu and Boerner 2021), coupling kinetochore and bridging microtubule fibers (Risteski et al. 2022) and being localized by dynein to spindle poles (Merdes et al. 2000). It is possible that there may be an as-yet undescribed interaction between NuMA and CENP-C in the kinetochore (NuMA has been reported to interact with kinetochore component Astrin (Chu et al. 2016)). Importantly, CENP-C, as a kinetochore component, is also important for chromosome alignment (Kwon et al. 2007). Thus, it is possible that here, the correlation analysis is revealing a role for both proteins in mitotic chromosome transactions even if both components are not directly interacting or localized in the same subcellular structure. NuMA has also been described to have a role in interphase nuclear structure (Merdes and Cleveland 1998) and has been proposed to be a part of nucleoskeleton. This could potentially explain, why it correlates with CENP-C together with nuclear envelope proteins.

The correlation between CENP-C and FIP1 is unexplained. FIP1 is reported to be a multivalent interaction scaffold for processing factors in human mRNA 3' end biogenesis (Muckenfuss et al. 2022). It is possible that there is an as-yet unknown interaction with CENP-C, but it is also possible that FIP1 is responsible for promoting the maturation of the mRNA of CENP-C, a key component involved with CENP-C and/or another component required for chromosome segregation. A previous study showing a surprising link between RNA processing components and sister chromatid cohesion turned out to be explained by the observation that the cohesin regulator sororin is a crucial rate-limiting factor whose RNA processing is essential for its normal accumulation and function (Sundaramoorthy et al. 2014).

### Correlation analysis suggests that CENP-E functions independently of the CCAN

We wished to determine whether mining published datasets could yield more fine-grained information about the functional interactions of proteins in specific pathways. To do this we looked at proteins involved in mitotic chromosome attachment to spindle microtubules. In vertebrate cells, two such pathways operate independently. One involves the complex set of proteins of the inner and outer kinetochore (CCAN, Mis12 and NDC80 complexes) The other involves CENP-E – a huge kinesin that despite its name is not a component of the CCAN. It is instead localized in the fibrous corona of the kinetochore (Cooke et al. 1997).

**Fig. 3:**
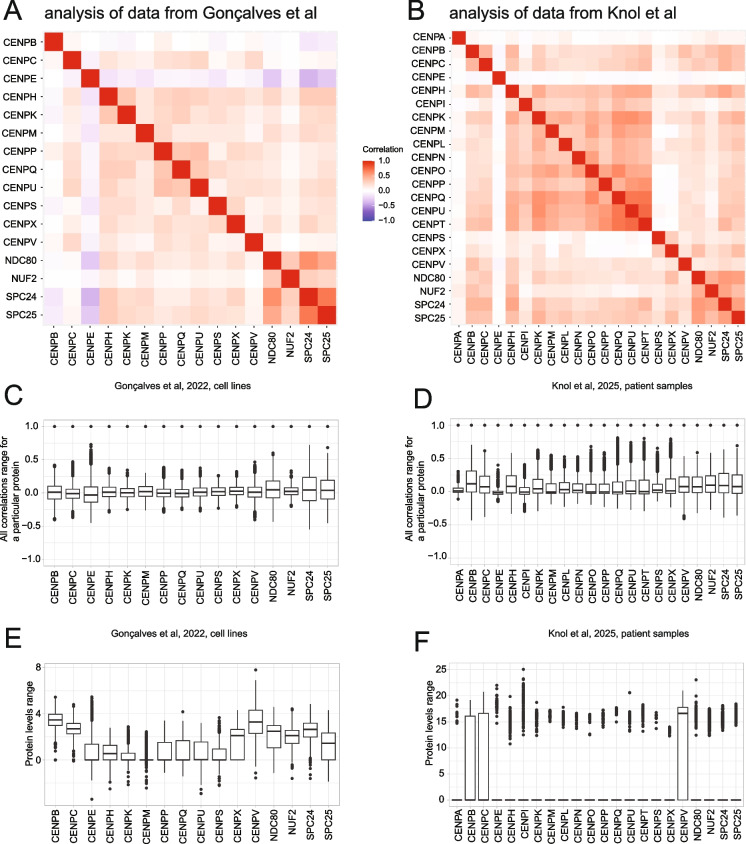
A, B. Correlation heatmap of levels of CENP proteins (excluding CENP-F and CENP-J) and NDC80 complex components present in the datasets from (Goncalves et al. 2022) and (Knol et al. 2025) respectively. C, D. Range of all correlations for the same selected proteins. Boxplots represent the median ± upper and lower quartiles. E, F. Range of abundance values for particular proteins. Boxplots represent the median ± upper and lower quartiles. In the (Knol et al. 2025) dataset, with the exclusion of CENP-B, -C and -V, an average of 1103 out of the 1172 samples lacked detectable levels of other centromeric proteins, resulting in median and the quartiles being set to 0

For this analysis, we plotted a heatmap displaying the correlation of known canonical CENPs present in the dataset (excluding CENP-F), as well as the NDC80 complex. This analysis is challenging, since data in these data-sets is sparse for centromeric proteins, nonetheless a simple correlation analysis was possible. While most centromere-associated network (CCAN) and NDC80 components displayed positive correlations with each other, CENP-E stood out in both datasets, displaying either no correlation or negative correlations with CCAN members present in the dataset as well as NDC80 (Fig. 3A, B). We confirmed that this did not occur because CENP-E had a consistently narrow range of correlations (Fig. 3C, D) or was expressed in the datasets at a low level compared to some CCAN and NDC80 components (Fig. 3E, F). Note that this difference in correlations is likely to be determined by the pathways in which CENP-E and the CCAN function during chromosome segregation and need not refer specifically to pathways of recruitment of CENP-E or the CCAN proteins to kinetochores.

CENP-E is a kinesin (Yen et al. 1992) that binds to unattached mitotic kinetochores and slides chromosomes to the spindle equator via its microtubule-motor activity (Yen et al. 1991; Kapoor et al. 2006; Craske and Welburn 2020). It is thought to be loaded to kinetochores mostly via interactions with BubR1 (Craske and Welburn 2020). Kinetochore component Nuf2 also interacts with CENP-E (albeit, not clear if directly) (Craske and Welburn 2020). Since CENP-E activity is presumed to be indispensable for chromosome alignment, it is somewhat surprising that cancer cell lines exhibiting high levels of CENP-E would show reduced levels of CCAN/NDC80 and vice versa, and that levels of CENP-E are not related to the levels of CCAN/NDC80 in the second analysed dataset. This analysis supports previous conclusions that CENP-E and the CCAN function in parallel pathways directing chromosome movements.

### Additional observations from correlation analysis

A strength of the analysis described here is that it can be applied to any protein abundant enough to be detected in a large number of datasets. This analysis can yield interesting and unexplained observations.

In the analysis of one dataset (Goncalves et al. 2022), we observed that mitotic perichromosomal layer protein Ki-67 (Booth and Earnshaw 2017; Remnant et al. 2021) exhibited mutual top-20 correlations with Aurora B, INCENP, Borealin and survivin – the four components of the chromosomal passenger complex (CPC) (Supplementary Table S2). The CPC is involved in regulating numerous chromosomal and mitotic spindle activities in mitosis, and interestingly, also present in the top 12 proteins correlated with Ki-67 were Tpx2, kinesin KIF22, topoisomerase IIα, RanGAP1 and RepoMan. We note that a previous study reported an association between the CPC and TPX2 (Iyer and Tsai 2012). To date no specific direct role for Ki-67 in mitosis involving microtubules has been reported (although a recent report suggested an indirect role (Hu et al. 2025)). Instead the protein has been proposed to be an essentially passive surfactant coating the chromosomes and responsible for formation of the perichromosomal layer (Booth et al. 2014; Cuylen et al. 2016; Stenstrom et al. 2020). That these Ki-67-correlating proteins might be correlated with one another is not surprising, as all are involved in chromosome segregation and mitotic spindle function. Although the factors driving the correlation remain undefined, we argue that the fact that these proteins rank in the top 12 of ~ 8500 proteins in the dataset cannot be ignored. At present, what this all has to do with Ki-67 remains a mystery and potentially a fruitful avenue for future research.

Of course, we cannot exclude that these proteins function in a common pathway in interphase, as heterochromatin is close to the nucleolar periphery, where Ki-67 is located. The fact that we observed this association only in the cancer cell line dataset, but not a second dataset mostly based on patient-derived samples, may reflect technical issues or could have biological meaning.

### Perspectives

The method described here takes a sustainable integrative cell biology approach (Earnshaw 2013) to generate hypotheses about functional biological relationships. Focusing here on the data mining stage, we present correlations that will require detailed biochemical follow-up to reveal underlying interactions and mechanisms. Our approach complements the use of databases including DepMap to find associated dependencies, in our case using protein-specific rather than gene-specific criteria. Our results demonstrate the power of using published resources to screen for novel associations and we would like to emphasize that re-analysing just two large-scale datasets offered a huge wealth of possibilities using a guilt-by-association strategy. Clearly, the range of databases analysed could be expanded to include any cell-based dataset that gives an accurate quantitation of protein levels in cells or tissues. As always in proteomics, missing values are a problem, so in expanding the analysis, it is critical that new datasets added to the analysis are comprehensive and do not just contain subsets of proteins.

One important limitation of this approach is the abundance and/or tissue specificity of the protein to be tested. In several instances, proteins that we wished to screen were detected in only a few of the samples. The analysis depends on having sample sizes sufficient for informative analysis. Another challenge posed by this analysis is that the results do not offer mechanistic explanations for the source of the correlations. Although we believe that the method reveals proteins that likely function in the same pathways, how or where they are functioning in those pathways is not revealed. We tried a very preliminary alphafold2 analysis to confirm selected top correlations based on (Goncalves et al. 2022) and also tried several physical pulldowns to look for interactions between proteins showing novel correlations. These results were not informative, but if we accept that proteins functioning in a single pathway may not co-localize or even be active at the same time, then this is not surprising.

We believe that many important future advances in biological sciences will result from sustainable collaborations between computational scientists, who can develop innovative new methods to mine existing databases to identify unexpected and novel hypotheses, working together with wet-lab scientists who can design, execute and interpret experiments that realise the mechanistic insights suggested by those hypotheses.

## Supplementary Information

Below is the link to the electronic supplementary material.

Supplementary file1 All correlations for centromeric proteins presented in Fig. 3. Cohesin and cohesin-related proteins are highlighted in yellow. The ranking was performed for CENP-C but could be changed for any presented protein accordingly (XLSX 6945 KB)

Supplementary file2 All correlations for Ki-67 and CPC. Ki-67 is highlighted in yellow. The ranking was performed for Ki-67 but could be changed for any CPC protein accordingly (XLSX 1677 KB)

## Data Availability

The code is available at GitHub via https://github.com/NatashaKochanova/Correlation-analysis-centromere.
